# Ileal mucosa-associated lymphoid tissue lymphoma presenting with small bowel obstruction: a case report

**DOI:** 10.1186/s13000-015-0353-6

**Published:** 2015-07-16

**Authors:** Zoe Kinkade, Olukemi A. Esan, Flavia G. Rosado, Michael Craig, Jeffrey A. Vos

**Affiliations:** Department of Pathology, West Virginia University, PO Box 9203, Morgantown, WV 26506 USA; Department of Medicine, Section of Hematology/Oncology, West Virginia University, PO Box 9162, Morgantown, WV 26506 USA

**Keywords:** Extranodal marginal zone lymphoma, MALT lymphoma, Maltoma, Ileum

## Abstract

Extranodal marginal zone lymphoma of mucosa-associated lymphoid tissue (MALT Lymphoma) of the gastrointestinal tract commonly involves the stomach in the setting of concurrent *Helicobacter pylori* (*H. pylori*) infection. Primary ileal MALT lymphoma is rare, and has not been associated with a specific infectious disease. We report a case of a 58-year-old man who presented to the emergency department with constipation and abdominal distension, and signs of an obstructing mass on computed tomography scan. A small bowel resection was performed which revealed an 8 cm saccular dilatation with thickened bowel wall and subjacent thickened tan-yellow tissue extending into the mesentery. Histologically, there was a diffuse lymphoid infiltrate consisting of small atypical cells with monocytoid features. These cells were CD20-positive B-lymphocytes that co-expressed BCL-2 and were negative for CD5, CD10, CD43, and cyclin D1 on immunohistochemical studies. Kappa-restricted plasma cells were also identified by in situ hybridization. The overall proliferation index was low with Ki-67 immunoreactivity in approximately 10 % of cells. No areas suspicious for large cell or high grade transformation were identified. The pathologic findings were diagnostic of an extranodal marginal zone lymphoma involving the ileum, with early involvement of mesenteric lymph nodes. Small hypermetabolic right mesenteric and bilateral hilar lymph nodes were identified by imaging. The bone marrow biopsy showed no evidence of involvement by lymphoma. The patient was clinically considered advanced stage and opted for therapy with rituximab infusions. After six months of therapy, follow-up radiologic studies demonstrated significant decrease in size of the mesenteric lymph nodes.

## Background

The gastrointestinal tract is involved by 30-40 % of all extranodal non-Hodgkin lymphomas [1]. The majority (60-75 %) of the extranodal lymphomas within the gastrointestinal tract involve the stomach and are associated with *Helicobacter pylori* (*H.pylori*) infection [1–3]. Involvement of other sites of the gastrointestinal tract by extranodal lymphomas has been reported including colon, jejunum, ileum and rectum [1, 2]. When lymphomas arise in the small bowel, they are most often diffuse large B-cell lymphomas; however, MALT lymphomas represent one third of the small bowel lymphomas [4, 5]. A variant of extranodal marginal zone lymphomas has been described in the duodenum known as immunoproliferative small intestine disease (IPSID) with a clinical manifestation of abdominal pain, diarrhea and malabsorption, which is prevalent in the middle East and Africa [6, 7]. Other extranodal lymphomas described in the small bowel are Burkitt lymphoma, mantle cell lymphoma, follicular lymphoma and T-cell lymphomas [4].

Extranodal marginal zone lymphoma typically arises in areas where mucosa-associated lymphoid tissue (MALT) has been acquired, but not necessarily where it is found under normal circumstances, such as the Peyer’s patches. In these acquired areas, lymphoma is believed to arise as a result of some chronic antigenic or inflammatory stimulus such as *H. pylori* gastritis or autoimmune process such as celiac disease, Sjogren syndrome, or Hashimoto’s thyroiditis [6]. Gastric cases associated with *Helicobacter pylori* infection may regress with antibiotic therapy [6, 8]. However, in other regions of the gastrointestinal tract antibiotic therapy has been found to be less effective [9]. Risk factors for small bowel lymphomas has been found to include immunodeficiency, inflammatory bowel disease and malabsorption syndromes [9]. In other sites, associations with other infectious agents, such as *Campylobacter jejuni* in the proximal small bowel, *Borrelia burgdorferi* in the skin, and *Chlamydia psittaci* in ocular adnexa, have also been described [6]. MALT lymphoma is an indolent lymphoma with an overall 5-year survival rate of 81 % [10]. It is a low-grade malignancy and is usually localized at the time of diagnosis. As such, patients often present at an early stage, even though they may have experienced and overlooked non-specific symptoms prior to diagnosis [11].

MALT lymphomas are thought to arise from the post-germinal center B-cells, normally found in the marginal zone of lymphoid follicles [12]. These malignant cells expand this zone and spill into the interfollicular areas, expanding beyond the dendritic meshwork of the follicle. In areas containing epithelial structures, the characteristic “lymphoepithelial lesions” are commonly present and characterized by eosinophilic degeneration of epithelial cells with influx of malignant lymphocytes [6]. The B-cells are generally small, with slightly irregular nuclei and moderate to abundant clear cytoplasm, accounting for the prototypical “monocytoid” appearance associated with this type of lymphoma. Plasmacytic differentiation may occur in a third of gastric MALT lymphomas [6]. By immunohistochemistry, the neoplastic cells express the pan-B-cell markers CD19, 20, 22, and 79a. Plasma cell differentiation can be demonstrated by CD 138 expression and concurrent light chain restriction. The dendritic cell meshwork are highlighted by CD21 and CD35, which may be helpful to demonstrate when the diagnosis of lymphoplasmacytic lymphoma is entertained, since, in such cases, a residual dendritic cell meshwork is not typically seen. CD5, CD10, and cyclin D1 are classically negative, aiding in differentiation of MALT from other low-grade B-cell lymphomas such as chronic lymphocytic lymphoma (typically CD5 positive), mantle cell lymphoma (typically CD5 positive), and follicular lymphoma (typically CD10 positive). Rarely, the malignant B-cells in marginal zone lymphoma may express CD5, posing a diagnostic challenge [6]. CD43 is positive in about half of the cases, and can help to differentiate a malignant proliferation (CD43 positive B-cells) from reactive MALT tissue [6]. Despite the above-described phenotypic features, there are no markers specific for marginal zone lymphoma. Due to this non-descript phenotype, a light-chain restricted B-cell population, negative for both CD5 and CD10, must be evaluated in conjunction with the morphologic findings to confirm the diagnosis.

The most common associated chromosomal abnormality in MALT lymphoma (particularly of gastric and pulmonary origin) is the t(11:18) (q21;q21) resulting in fusion of the API2 and MALT1 genes, occurring in approximately 50 % of cases [6, 12]. Cases that are positive for t(11:18) (q21;q21) tend to not regress following therapy directed at eradicating *H. pylori*. Other translocations associated with extranodal marginal zone lymphoma include t(1;14) (p22;q32), t(3:14) (p14;q32), and t(14;18) (q32;q21) [6]. Other non-specific trisomies have been reported in extranodal marginal zone lymphomas [6].

While extranodal marginal zone lymphoma of mucosa-associated lymphoma tissue (i.e., MALT lymphoma) is one of the more common primary gastrointestinal lymphomas, a review of the literature shows very few reported cases of primary MALT lymphoma involving the ileum, occurring predominantly in Asian patients [1, 4, 10]. The authors herein present a case of primary ileal MALT lymphoma presenting with bowel obstruction in a Caucasian adult.

## Case presentation

A 58-year-old Caucasian male presented to the emergency department with complaints of constipation and abdominal distension. He had experienced nausea and decreased bowel movements for a week, which were not alleviated by stool softeners or enemas. There was no history of diarrhea, fever, chills, weight changes, night sweats, hematochezia, or melena. A colonoscopy 4 years prior had only revealed a small benign polyp. The patient otherwise had no significant past medical history and was not taking any medications. No history of abdominal surgeries or significant family history of cancer was elicited. He denied the use of alcohol or smoking, but admitted to using smokeless tobacco. Vital signs and physical exam were essentially unremarkable. The abdomen was not visibly distended and normal bowel sounds were present. No hepatosplenomegaly or lymphadenopathy was appreciated. Laboratory results showed normal complete blood count, coagulation parameters, electrolytes, liver function tests, pancreatic enzymes, and kidney function. Alpha fetoprotein (AFP) and carcinoembryonic antigen (CEA) were within normal limits. An abdominal computed tomography scan (CT scan) was concerning for a neoplasm causing small bowel obstruction, showing mid to distal small bowel dilatation with abnormal wall thickening and significant retained debris. The patient underwent a laparotomy and resection of a distal segment of ileum was performed.

**Fig. 1:**
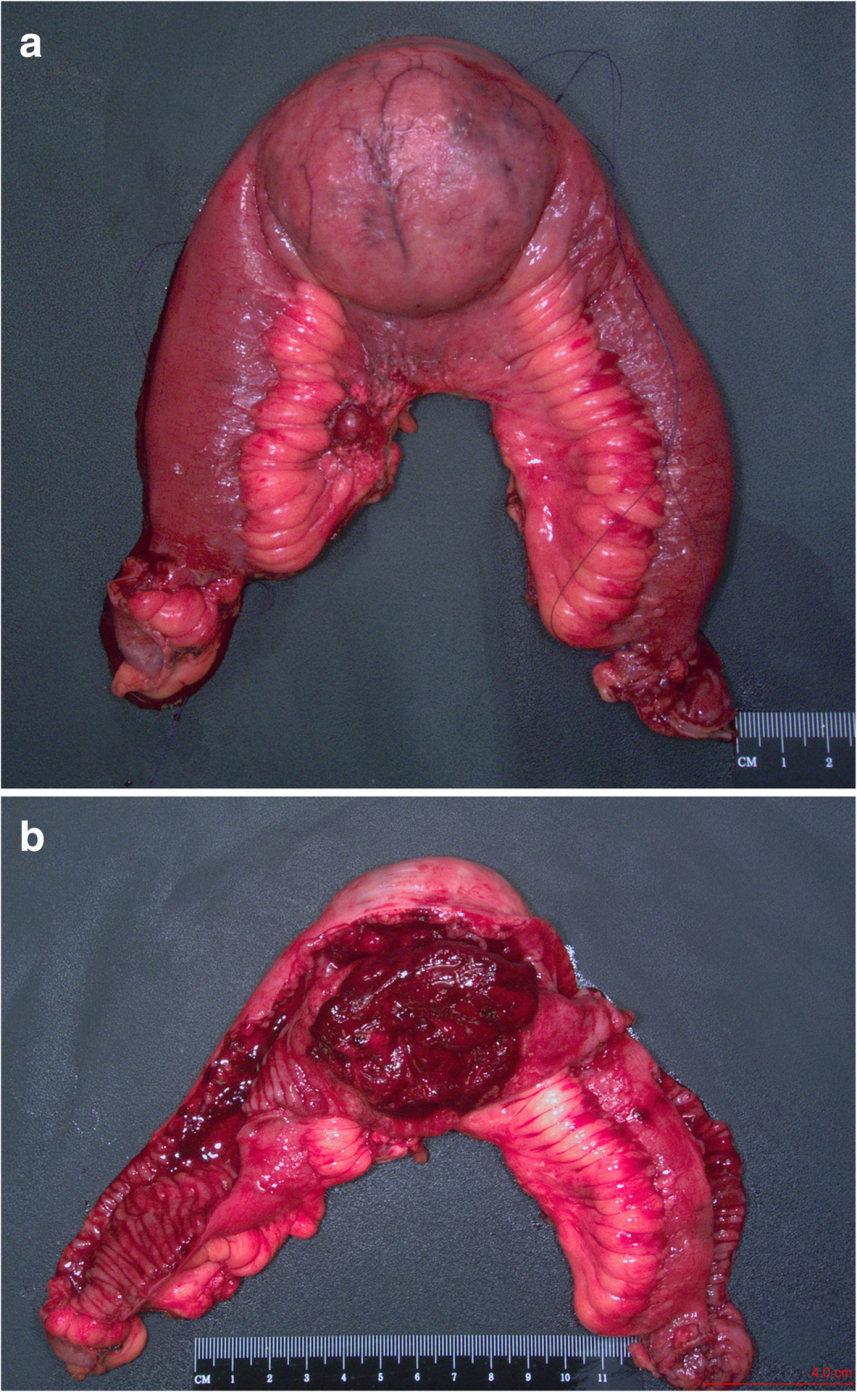
**a** Gross specimen, distal ileum with saccular dilatation. **b** Gross specimen, ileum, incised to show hemorrhagic mucosa within saccular dilatation

**Fig. 2:**
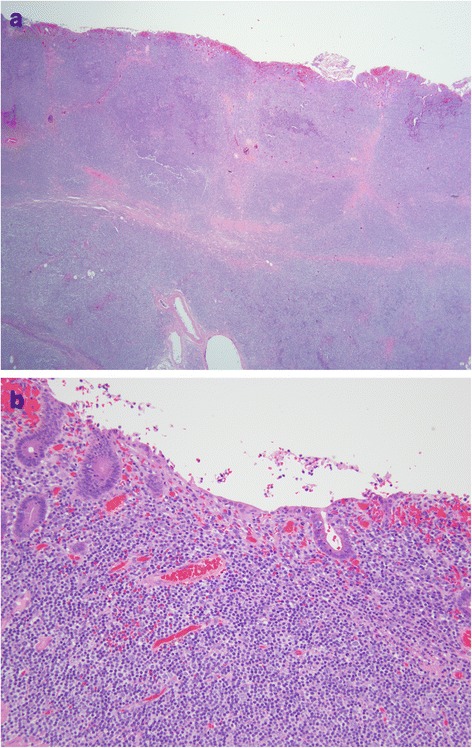
**a** Ileal wall with transmural nodular lymphocytic infiltrate. H&E. 20x. **b** Lymphoplasmacytic infiltrate with monocytoid features. H&E. 200x

**Fig. 3:**
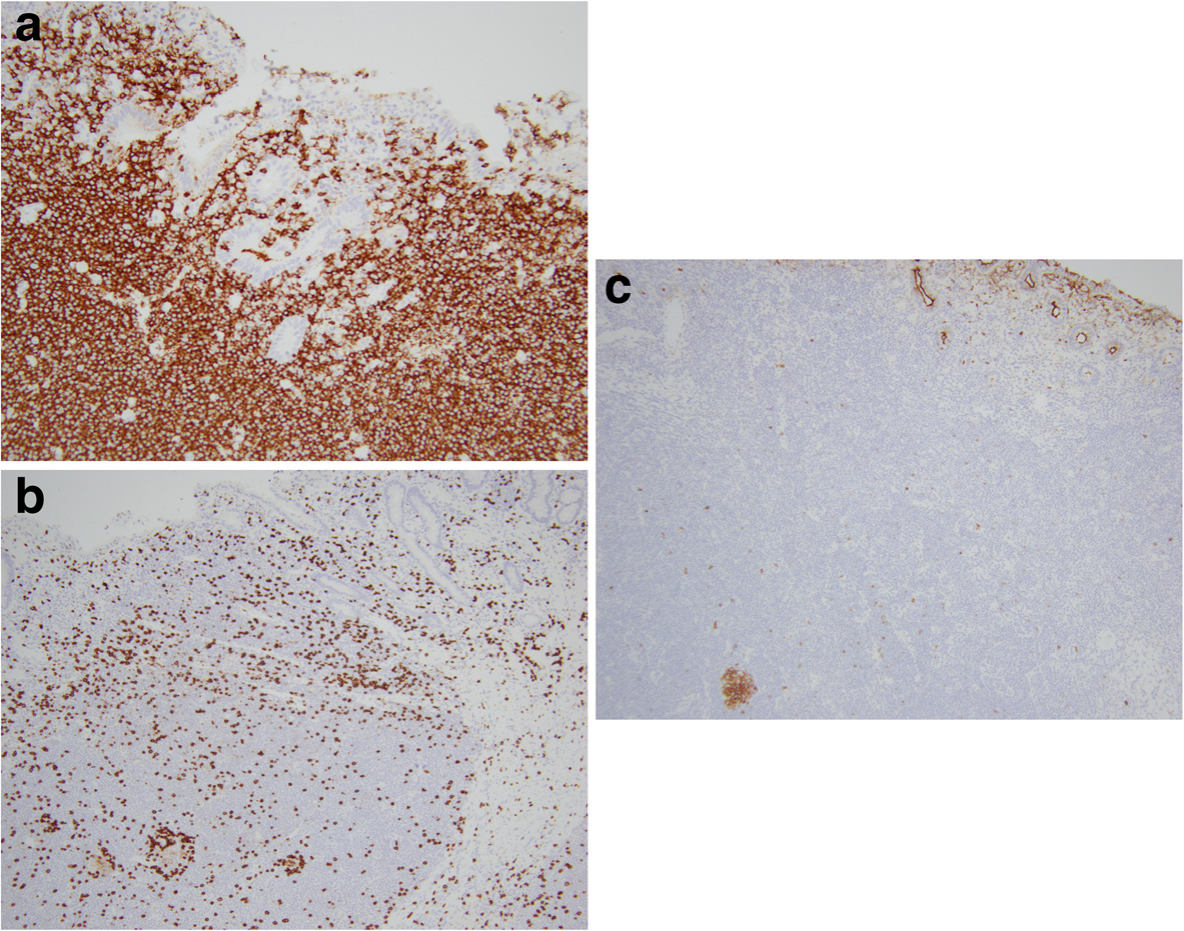
**a** Lymphocytic infiltrate consists predominantly of CD20 positive B-cells. CD20 immunohistochemical stain. 200x. **b** B-cells are negative for CD5 which highlights scattered T-cells. CD5 immunohistochemical stain. 200x. **c** B-cells are predominantly negative for CD10 which highlights few residual germinal center B-cells. CD10 immunohistochemical stain. 200x

**Fig. 4:**
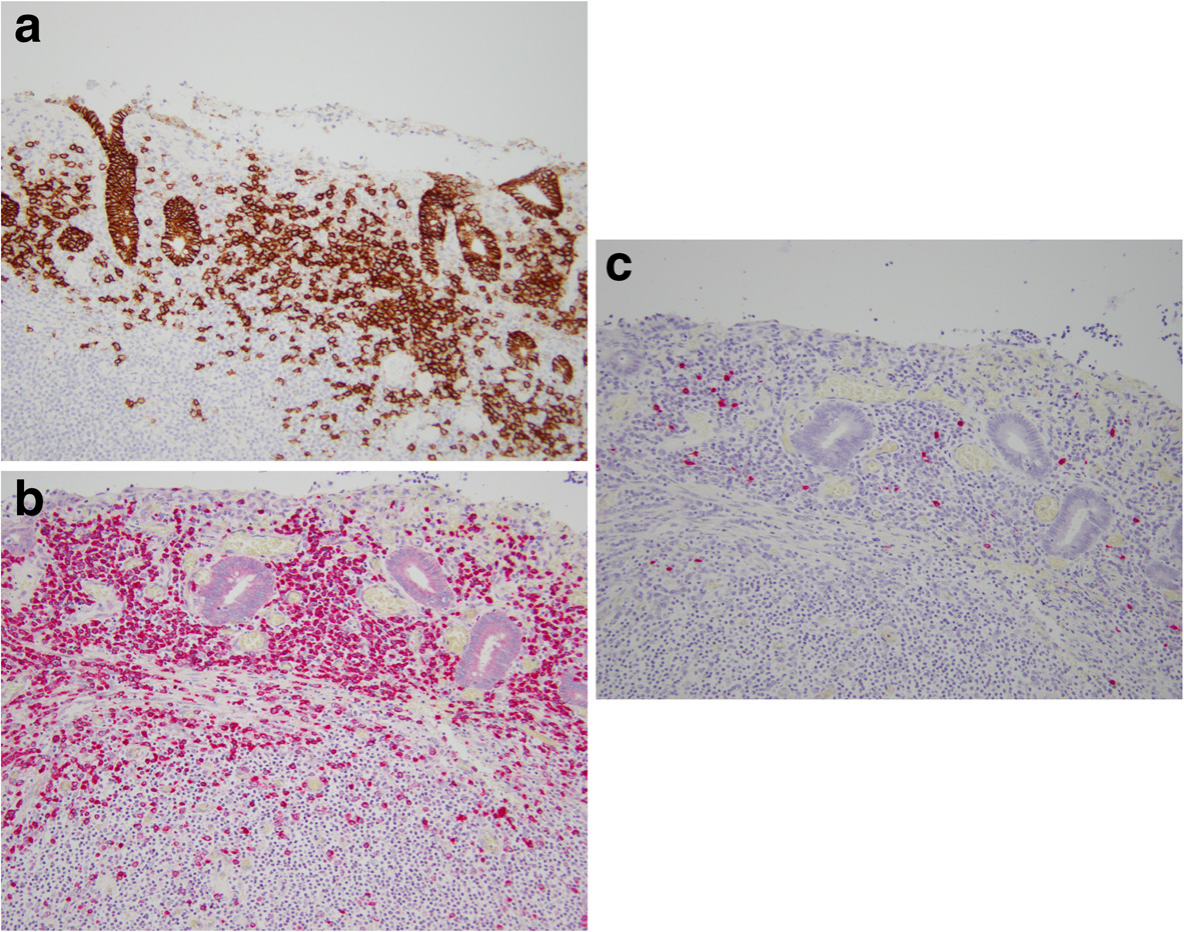
**a** Clusters of plasma cells located near the mucosal surface. CD138 immunohistochemical stain. 200x. **b** Plasma cells with predominantly kappa light chain. Kappa in situ hybridization. 200x. **c** Rare plasma cell with lambda light chain. Lambda in situ hybridization. 200x

**Fig. 5:**
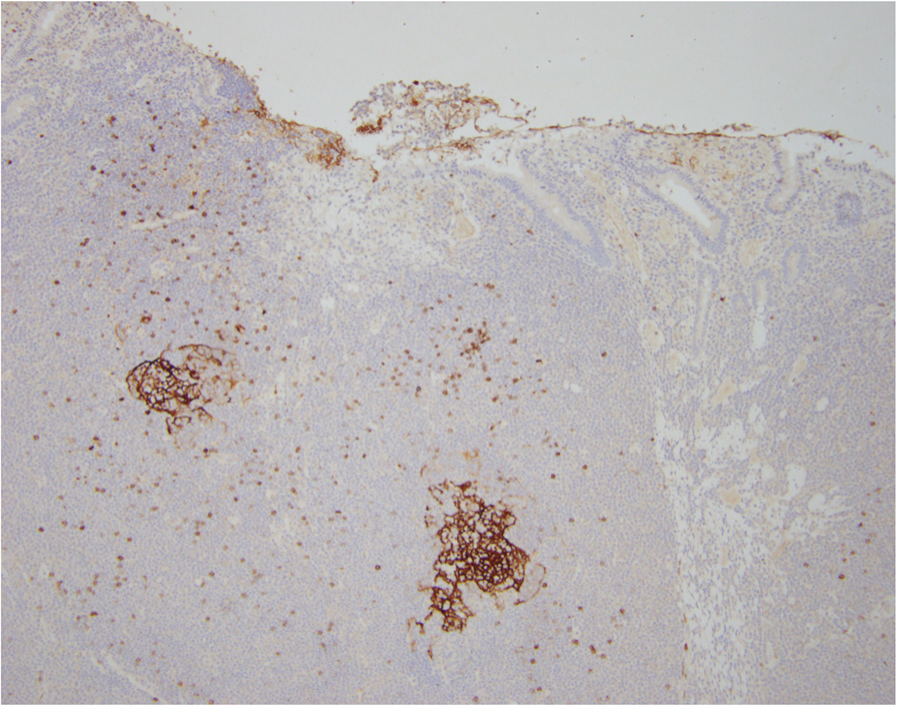
Residual dendritic meshwork identified within lymphocytic infiltrate. CD23 immunohistochemical stain. 100x

**Fig. 6:**
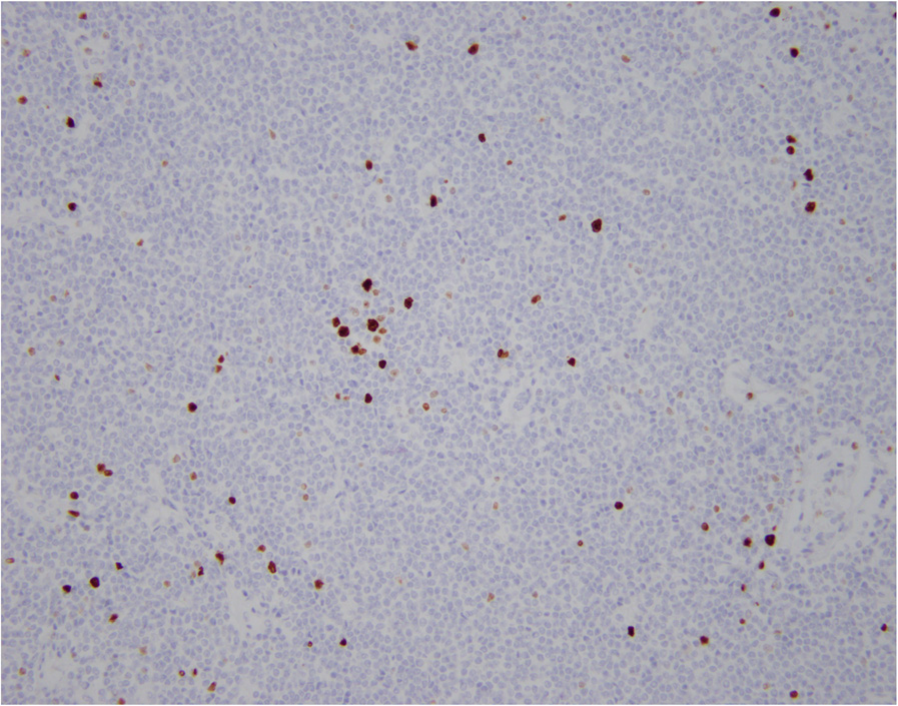
Lymphocytic infiltrate with low proliferation index. Ki-67 immunohistochemical stain. 200x

On gross examination, a 39 cm portion of distal ileum was received and the central portion contained an 8 cm length saccular dilatation that had a hemorrhagic and nodular mucosa (Fig. 1a and b). The bowel wall measured up to 0.9 cm in thickness and demonstrated underlying, ill-defined pale yellow tissue extending into the mesentery. On microscopic examination, an extensive transmural atypical nodular and diffuse lymphoid infiltrate was present and consisted predominantly of small B-lymphocytes, few plasma cells and focal areas of monocytoid B-cells (Fig. 2a and b). This lymphoid infiltrate extended into the subserosal adipose tissue. The majority of nodules did not contain germinal centers; however, few scattered small germinal centers were noted. Overlying the atypical lymphoid infiltrate, villous structures of the intestinal epithelium was flattened and focally denuded with sparse epithelial crypts and few lymphoepithelial lesions. No foci of necrosis were identified. No areas suspicious for large cell or high grade transformation were identified. The uninvolved mucosa was unremarkable with normal villi and crypt architecture. The atypical lymphoid cells were positive for CD20 (Fig. 3a) and BCL-2, while negative for CD5 (Fig. 3b), CD10 (Fig. 3c), CD43, and cyclin D1 by immunohistochemistry. The CD138 positive plasma cells (Fig. 4a) were most numerous and clustered beneath the mucosal surface and were kappa-restricted by light chain in situ hybridization studies (Fig. 4b and c). The residual dendritic meshwork within lymphoid nodules was identified by CD23 staining (Fig. 5). The proliferation rate of the lesion was low, with a Ki-67 percentage of approximately 10 % (Fig. 6). The overall morphologic and immunophenotypic features are consistent with a diagnosis of marginal zone lymphoma. Few prominent lymph nodes were identified in the mesentery (measuring up to 1.5 cm in greatest diameter). Microscopic examination showed partial effacement of these mesenteric lymph nodes with germinal center infiltration by BCL-2 positive B-cells, consistent with partial nodal involvement by marginal zone lymphoma.

The large ileal mass in the setting of small mesenteric and hilar lymph nodes was clinically consistent with a diagnosis of extranodal marginal zone lymphoma arising from the ileum. A complete staging evaluation including PET and CT imaging studies revealed additional small hypermetabolic mesenteric and bilateral hilar lymph nodes. No other lesions were noted in the stomach, thyroid, liver, pancreas or other organs. A staging bone marrow biopsy was negative for involvement by lymphoma, by morphologic and flow cytometric analysis. The patient was clinically staged as 3E (lymphadenopathy above and below the diaphragm). Treatment was initiated with single agent rituximab (monoclonal antibody against CD20) every other month, and is planned to continue for 2 years. After six months of treatment, the patient was asymptomatic and radiologic studies demonstrated significant decrease in size of the mesenteric lymph nodes.

## Discussion

Small bowel obstruction in industrialized countries is usually due to adhesions, which account for approximately 70 % of cases [13]. Other causes of small bowel obstruction are malignancies, inflammatory bowel disease and hernias. Pre-op detection of small bowel malignancies is improved with the introduction of new sophisticated endoscopic techniques (e.g. capsule endoscopy). However, due to non-specific clinical presentations, diagnosis in many patients is delayed [4].

Diffuse large B-cell lymphoma is the most common primary gastrointestinal tract lymphoma, with the small bowel as the site of origin 15–30 % of the time [5]. MALT lymphoma of the small bowel is rare. The differential diagnosis of MALT lymphoma in this site includes immunoproliferative small intestinal disease (IPSID), an alpha heavy chain disease that usually occurs in the proximal small intestine (duodenum), and shows prominent plasmacytic differentiation without demonstrable light chain expression [6, 7]. IPSID has been associated with *Campylobacter jejuni* infection [7] rather than with *Helicobacter pylori* infections seen in gastric MALT lymphomas. This is an unusual case in which extranodal marginal zone lymphoma presented as ileal obstruction, in a patient with no known history of a chronic inflammatory or autoimmune disease.

## Conclusions

In conclusion, this case represents a rare case of extranodal marginal zone lymphoma involving the ileum presenting as small bowel obstruction. Further studies are required to understand the etiology of ileal MALT lymphomas, with possible identification of causative agents and better understanding of pathogenesis of this malignancy. Eradication of treatable infectious organisms may help in preventing the occurrence of ileal MALT lymphomas in the future. Appropriate identification of these cases is important for early intervention, and proper management that includes targeted agents and chemotherapy, with possible avoidance of surgical procedures.

## Consent

Written informed consent was obtained from the patient for publication of this case report and accompanying images. A copy of the written consent is available for review by the Editor-in –chief of this journal.
